# Semiconductor Characterization by Terahertz Excitation Spectroscopy

**DOI:** 10.3390/ma16072859

**Published:** 2023-04-03

**Authors:** Arūnas Krotkus, Ignas Nevinskas, Ričardas Norkus

**Affiliations:** Centre for Physical Sciences and Technology, Saulėtekio al. 3, 10257 Vilnius, Lithuania; ignas.nevinskas@ftmc.lt (I.N.); ricardas.norkus@ftmc.lt (R.N.)

**Keywords:** terahertz, THz emission, THz excitation spectroscopy, ballistic electrons, heterojunction offset, bismuth layer, nanowires, band structure, subsidiary valleys, anisotropic photocurrent

## Abstract

Surfaces of semiconducting materials excited by femtosecond laser pulses emit electromagnetic waves in the terahertz (THz) frequency range, which by definition is the 0.1–10 THz region. The nature of terahertz radiation pulses is, in the majority of cases, explained by the appearance of ultrafast photocurrents. THz pulse duration is comparable with the photocarrier momentum relaxation time, thus such hot-carrier effects as the velocity overshoot, ballistic carrier motion, and optical carrier alignment must be taken into consideration when explaining experimental observations of terahertz emission. Novel commercially available tools such as optical parametric amplifiers that are capable of generating femtosecond optical pulses within a wide spectral range allow performing new unique experiments. By exciting semiconductor surfaces with various photon energies, it is possible to look into the ultrafast processes taking place at different electron energy levels of the investigated materials. The experimental technique known as the THz excitation spectroscopy (TES) can be used as a contactless method to study the band structure and investigate the ultrafast processes of various technologically important materials. A recent decade of investigations with the THz excitation spectroscopy method is reviewed in this article. TES experiments performed on the common bulk A3B5 compounds such as the wide-gap GaAs, and narrow-gap InAs and InSb, as well as Ge, Te, GaSe and other bulk semiconductors are reviewed. Finally, the results obtained by this non-contact technique on low-dimensional materials such as ultrathin mono-elemental Bi films, InAs, InGaAs, and GaAs nanowires are also presented.

## 1. Introduction

Ultra-short electrical pulses generated employing femtosecond laser beams, the application of which became widespread after the pioneering works of D. Auston [1] and D. Grischkowsky [2], are now widely used to characterize the physical properties of various materials, including semiconductors. Pulses with a duration of approximately half of the THz wave period are now routinely used in such techniques as THz time-domain spectroscopy (THz-TDS), optical-pump THz-probe (OPTP), and THz excitation spectroscopy (TES).

Most of the materials irradiated by femtosecond laser pulses generate THz radiation transients. They are generated from various metals [3], gases [4], and liquids [5]. This effect is especially strong and nearly universal in semiconductor materials; the emission of THz pulses from semiconductors can be caused by several different physical processes. Therefore, research on surface THz emission from semiconductors has received special attention. This phenomenon was first observed in InP and a variety of other semiconductor crystals by X.-C. Zhang et al. [6]. A subsequent paper of the same group [7] provided a more detailed analysis of this phenomenon, comparing results of various materials: Ge, Si, binary and ternary compounds of groups A3B5, A2B6, and superlattices composed of hem.

The initial experiments were performed using a CPM dye laser generating 650 nm wavelength femtosecond pulses, with the Ti:sapphire laser (λ = 800 nm) later becoming the main research tool. New research possibilities opened up when optical parametric amplifiers (OPA) were used in surface THz emission studies. They made it possible to obtain femtosecond optical pulses with wavelengths covering a wide spectral range and, therefore, study the dynamics of electrons excited at various initial energies within materials. Among the results obtained using femtosecond OPA pulses, one can distinguish the energy determination of additional conduction band valleys in semiconductors [8] and the line-up of energy bands in semiconductor heterojunctions [9].

In this article, we review the generation of THz pulses in semiconductors and semiconductor structures excited by femtosecond optical pulses of various wavelengths-a method known as the TES. At first, the most important physical mechanisms of this effect is presented, while examining in detail one of them-the photocurrent created by ballistically moving charge carriers. Later, the results of research on various semiconductor materials (cubic crystals of the A3B5 group, materials with hexagonal symmetry, two-dimensional structures, nanowires, quantum dots, etc.) are described. Finally, we discuss other various possibilities and perspectives for the use of TES in material science.

## 2. Mechanisms of Surface THz Emission

**Figure 1:**
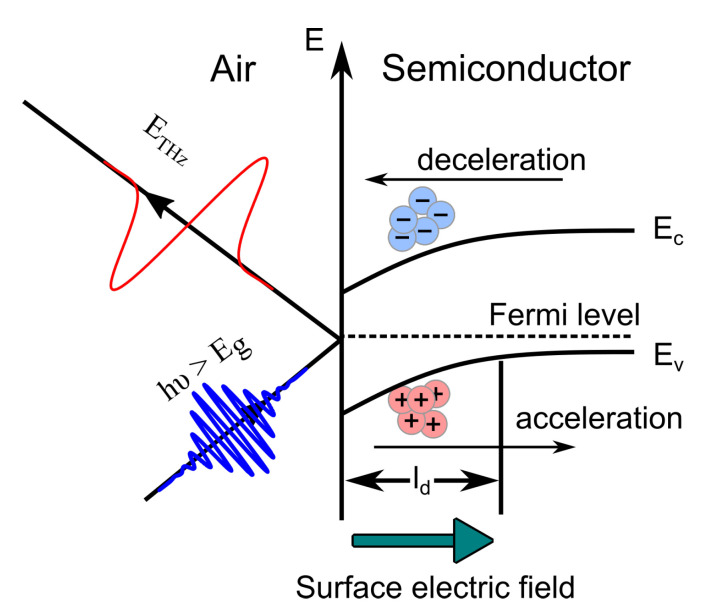
Band diagram of a p-doped semiconductor. Surface states near the conduction-band edge causes Fermi level “pinning” at the interface. Photocarriers are swept across the depletion width $l_{d}$ by the built-in field.

In the first works devoted to THz emission from semiconductor surfaces [6,7], this phenomenon was explained in terms of a dynamic electric dipole, which is formed when the surface electric field arising from surface states spatially separates electrons and holes created by light (Figure 1). The free carriers driven by the static electric field at the surface accelerate along the field direction and form a transient photocurrent. The rise time of the photocurrent pulse is comparable to the optical pulse duration, while the fall time is limited by the transit time of the carriers across the field region (assuming the transit time is shorter than the carrier lifetime). The fast time-varying current J(t) radiates electromagnetic waves. In the far field, the amplitude of the radiation $E_{THz}$ is proportional to the charge acceleration or the time derivative of the photocurrent

(1) $$E_{THz}\propto \frac{J(t)}{dt}$$

Therefore, the authors of ref. [7] proposed to use their discovered phenomenon as a contactless method for determining the built-in electric field at a semiconductor surface. As femtosecond lasers operating at longer wavelengths and smaller photon energies, namely, Yb-doped potassium gadolinium vanadate (Yb:KGW) laser (hν≈ 1 eV) and Er-doped fibre laser (hν≈ 0.8 eV) are used in the experiments, it becomes clear that not all results can be explained by this simple dynamic dipole created by the surface electric field model. One problem is related to THz emission from the narrow-gap semiconductors such as InSb or InAs. Although the surface field in such semiconductors is generally weak, it turns out that they can be more efficient THz emitters than materials with a wider energy bandgap. On the other hand, the sole surface electric field effect makes it difficult to explain the emitted THz pulse amplitude dependencies on the angle between laser polarization and the crystal axes (the azimuthal angle dependencies).

**Figure 2:**
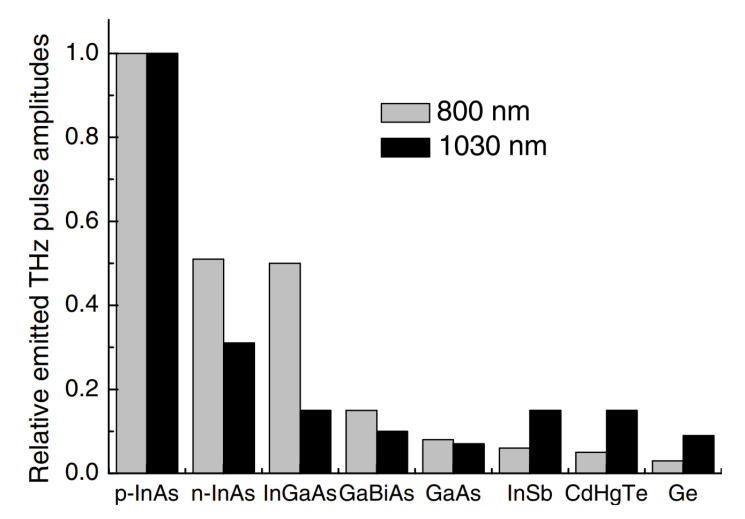
Comparison of THz pulse amplitudes emitted from several semiconductor surfaces after their excitation by femtosecond optical pulses of two different wavelengths. Reproduced with permission from [12] © IOP Publishing.

**Figure 3:**
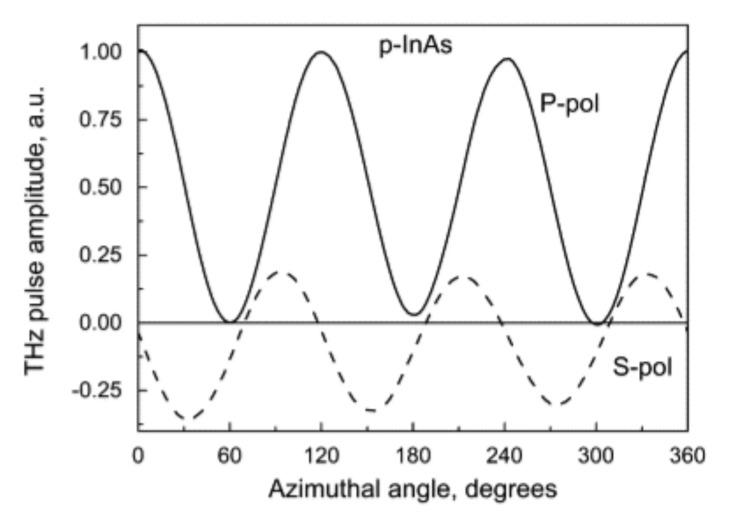
Azimuthal angle dependencies of THz pulse amplitude from (111)-cut surfaces of p-InAs after femtosecond pulse excitation with P- (full lines) and S-polarized (dashed lines) light [10].

Both of these circumstances are illustrated in Figure 2 and Figure 3 [10]. Figure 2 compares the amplitudes of THz pulses generated from various semiconductor surfaces illuminated by femtosecond Yb:KGW and Ti:Sapphire lasers. A moderately p-doped InAs crystal (hole concentration from 10^16^ cm^−3^ to 10^17^ cm^−3^) is up to date the most efficient surface THz emitter [11] at all three femtosecond laser wavelength ranges mentioned above. Figure 3 shows the THz pulse amplitude azimuthal angle dependencies from a p-type InAs crystal surface cut along the (111) crystal plane.

The explanation to the first of those observations was proposed by T. Dekorsy et al. who studied the emission of THz radiation from narrow-gap ($\epsilon_{g}=$ 0.34 eV) tellurium crystals [13]. It was assumed in his paper that the THz emission from tellurium can be explained by the ultrafast buildup of the photo-Dember field within the first 100 fs after optical excitation. The Dember field results from different diffusion coefficients of electrons and holes. In the tellurium crystal, electron mobility is approximately two times larger than the hole mobility, therefore, electrons can travel further from the illuminated surface into the bulk of the crystal than holes, creating a fast-changing electric dipole. In such crystals as InSb or InAs this ratio is much larger—close to a hundred, thus the buildup of the photo-Dember field became recognized as the main source of broadband THz emission from these semiconductors.

On the other hand, both of the physical mechanisms mentioned above, the surface photocurrent surge and the photo-Dember effect, rely on electrical conductivity, and in cubic semiconductors such as A3B5 compounds, the conductivity is isotropic. Therefore, these mechanisms were not sufficient to explain the azimuthal angle dependencies of the emitted THz signal. Nevertheless, it was observed [14] that in the studied A3B5 semiconductors, these dependencies had the same symmetry as the nonlinear optical response described by the equation

(2) $$\vec{P}\left(t\right)=\chi^{\left(2\right)}{\vec{E}}^{2}\left(t\right)+\chi^{\left(3\right)}{\vec{E}}^{2}\left(t\right)\vec{F}$$

where $\chi^{\left(2\right)}$ and $\chi^{\left(3\right)}$ are the second and third-order optical susceptibilities that determine the intensity of such nonlinear effects as optical rectification (OR) and electric field-induced optical rectification (EFIOR), respectively, E(t) and *F* are optical and dc electric fields. The THz emission caused by these phenomena when excited with optical pulse wavelengths corresponding to the transparency range of the material is well described using the values of nonlinear optical susceptibilities [14]. However, to explain the experimental results of THz measurements performed with shorter than the absorption edge wavelength optical pulses, it was necessary to introduce significantly higher values of these parameters than those observed at photon energies lower than $\epsilon_{g}$, by approximately 3 orders of magnitude for GaAs and InP [14] and even more for InSb and InAs [15]. Therefore, the microscopic mechanism of these high optical susceptibility values remained unclear. The description of THz pulse generation using the so-called photo-Dember mechanism is also not completely correct. The THz pulse amplitude reaches its maximum within 100–200 fs after semiconductor photoexcitation. Since this is less than the momentum and energy relaxation times of typical A3B5 semiconductors, ~200 fs and ~1 ps respectively, the mobilities and diffusion coefficients of electrons and holes do not reach their stationary values yet, so various hot carrier effects must be taken into account.

The two main effects of hot electrons in semiconductors on sub-pico and picosecond time scales are the ballistic motion at times shorter than momentum relaxation duration and the drift velocity overshoot at later times. M. Tonouchi [16] proposed a simple formula to estimate a THz pulse resulting from the photocurrent caused by ballistic motion of photoexcited electrons. That current appears throughout the laser pulse duration and it is proportional to the product of photoelectron density Δn and their group velocity. The first of these quantities is proportional to the laser pulse intensity $I_{p}$, while the second to the electron quasi-momentum $k\approx \sqrt{\epsilon_{ex}/m_{n}}$, where $\epsilon_{ex}$ is the electron excess energy, and $m_{n}$ is its effective mass. By inserting these values, we get that the amplitude of a THz pulse is proportional to

(3) $$E_{THz}\propto I_{p}\sqrt{\frac{\epsilon_{ex}}{m_{n}}}$$

**Figure 4:**
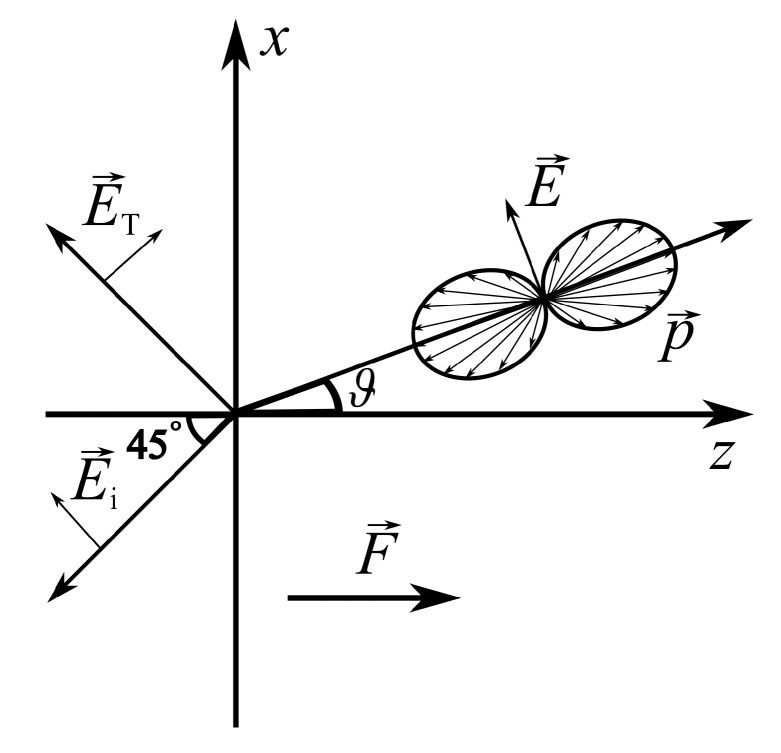
The optical alignment of electrons excited from heavy-hole subband by linearly polarized light. ${\vec{E}}_{i}$ is the incident electric field, ϑ is the refraction angle, $\vec{E}$ is the electric field of refracted light, ${\vec{E}}_{T}$ is the THz electric field radiated in quasi-reflection direction. Reproduced from [17], with the permission of AIP Publishing.

A microscopic theory of THz radiation emission from InAs and InSb crystal surfaces was proposed by V. L Malevich et al. [17]. In this work it was shown that, in general, the photoconductivity in zinc blende type semiconductors can be characterized by the same dependencies on the electric field orientation as the OR or EFIOR effects. In [17], the anisotropy of THz emission from narrow-gap A3B5 semiconductors was explained by the anisotropic photoconductivity caused by the nonparabolicity and nonsphericity of the electron energy dispersion law together with the optical alignment of electron momenta during the photoexcitation. The optical orientation of photoexcited electrons is related to the selection rules of light-induced transitions. In the case of linearly polarized light, it causes an anisotropic distribution of photoelectron momenta (Figure 4). In cubic A3B5 group semiconductors, transitions from the heavy-hole band create electrons whose momenta are mostly oriented parallel to the light beam propagation direction, while transitions from the light-hole band are perpendicular to that direction [18]. In the case of stationary photoexcitation, this phenomenon is weak and can only be observed at cryogenic temperatures and at electron excess energies lower than optical phonon energy. Under such conditions, a polarized photoluminescence [19] and lateral photocurrents [20] in GaAs were documented. The transient photocurrent occurring in semiconductors after femtosecond laser pulse excitation, which generates a THz pulse within 100–200 fs—a time shorter than the momentum relaxation, the optical momentum alignment effect is not lost even at room temperatures. Therefore, the speed of ballistically moving electrons and their travelling direction is dependant on the unevenly distributed effective mass arising from the non-parabolic nature of conduction band dispersion law. The pronounced deviation from spherical dispersion may lead to significant effective mass anisotropy. For the same reason, the optical momenta alignment of electrons can create a photocurrent component parallel to the illuminated crystal surface.

**Figure 5:**
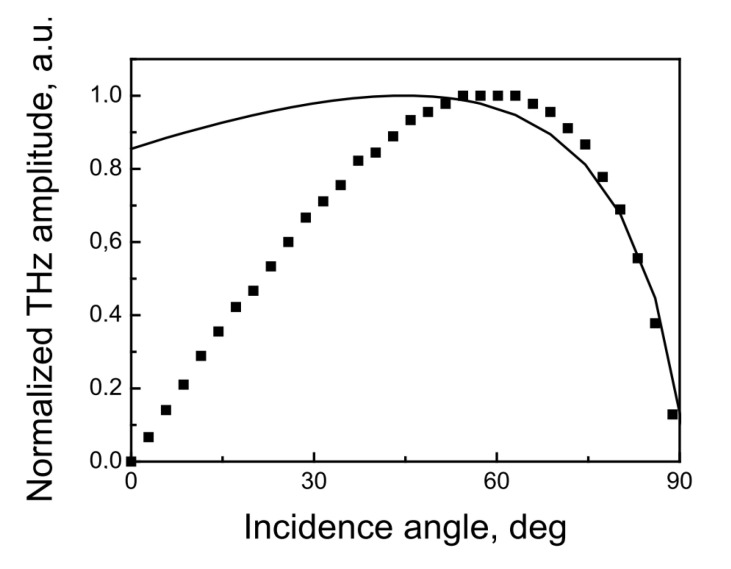
Calculated THz field dependencies on optical beam incidence angle due to the lateral photocurrents Equation (4) (full line) and the photo-Dember effect (points). Reprinted with permission from [21] © Optica Publishing Group.

The presence of lateral photocurrents was confirmed by studies of THz emission from InAs [21] and InSb [22] crystals that were excited by a laser beam perpendicular to their surfaces. First of all, this is confirmed by the very presence of a THz signal in transmission geometry, which, for example, is not possible in the photo-Dember effect case (Figure 5). According to the Equation 4 from ref. [17], the THz electric field (p polarization) radiated from the (111) surface illuminated at an angle ϑ to its normal is proportional to

(4) $$E_{THz}^{p}\propto \left(\alpha +\frac{\gamma }{3}+\beta \sin^{2}\vartheta \right)\sin\vartheta -\cos^{2}\vartheta \left[\left(\beta +\frac{2}{3}\gamma \right)\sin\vartheta +\frac{\gamma }{3\sqrt{2}}\cos\vartheta \cos3\phi \right]$$

where α, β, γ are coefficients expressed in terms of nonvanishing third-order nonlinear photocurrent tensor components as $\alpha =\sigma_{zzxx}$, $\beta =2\sigma_{zxxz}$, $\gamma =\sigma_{zzzz}-\sigma_{zzxx}-2\sigma_{zxxz}$, ϑ is the angle of the refracted optical beam in the sample, and ϕ is the azimuthal angle.

**Figure 6:**
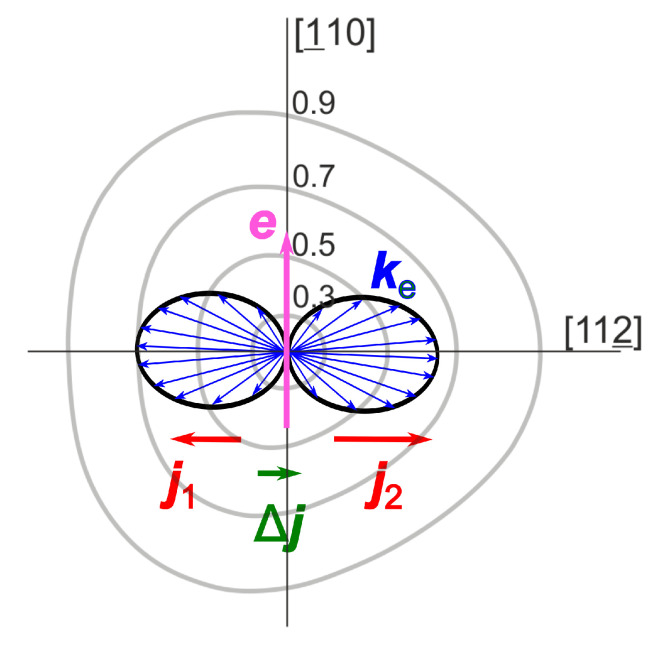
Cross sections of the conduction band isoenergetic surfaces at different excess energies (grey lines) and the momentum distribution of photoelectrons excited form heavy-hole valence band (eight shaped curve). *e* is the optical polarization vector, Δj is the uncompensated lateral photocurrent. Reprinted with permission from [22] © Optica Publishing Group.

The appearance of lateral photocurrent is illustrated in (Figure 6, which shows the isoenergetic surface of the conduction band of (111) oriented InSb together with the momentum distribution of photoelectrons excited from heavy-hole valence band. Electrons moving to the right have smaller effective masses than those moving in the opposite direction, therefore their contributions are not balanced and the total photocurrent shall flow in the $\left[11\bar{2}\right]$ direction. Because the isoenergetic surface has a trigonal symmetry for (111) crystalline plane, the azimuthal angle dependence of the lateral current is proportional to cos3ϕ, as it has been observed experimentally in [22].

## 3. THz Excitation Spectroscopy of A3B5 Semiconductors

The first spectral measurements of surface THz emission were performed by R. Adomavičius et al. on InSb and InAs crystals [8]. The THz excitation spectra for both of these materials were non-monotonic, with well-defined maxima. These maxima were explained by electron scattering into high effective mass subsidiary valleys of the conduction band. Because relatively high power and low repetition rate (1 kHz) pulses from an optical parametric amplifier were used, the measurements were performed only at high excitation levels, which made interpretation of the results difficult.

**Figure 7:**
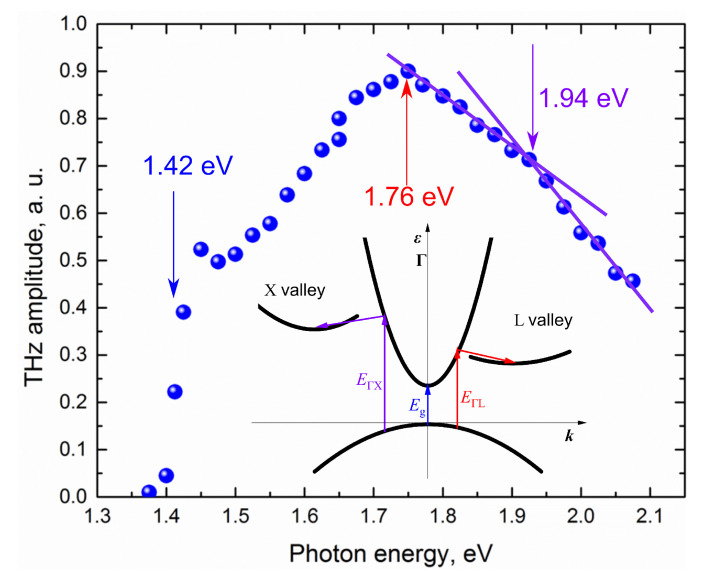
TES spectrum of SI-GaAs (blue dots) when THz pulse amplitudes are normalized to a constant photon number. Additional blue, red and purple arrows show energies corresponding to band structure parameters. The inset shows a schematic GaAs band structure.

OPA Orpheus (Light Conversion Ltd.) the main research tool in later experiments of this group is better suited for TES. The wavelength of OPA output can be changed in a wide range from 2.6 μm to 0.6 μm, and the 200 kHz repetition rate of 140–160 fs duration pulses generated by it is more suitable for the lock-in amplifier. THz excitation spectra of several A3B5 group semiconductors measured with such a system are presented in [23]. These spectra exhibit the sharp maxima as in previous InAs and InSb crystal studies. We shall examine their occurrence in more detail in the case for GaAs. Figure 7 shows the THz excitation spectrum of semi-insulating GaAs, while the inset depicts GaAs’s energy band diagram. Three characteristic photon energies are marked in the diagram and spectrum. hν= 1.42 eV, at which the THz emission starts, corresponds to the energy bandgap of the material. The main maximum of the spectral dependence at hν= 1.76 eV corresponds to the onset of electron transfer from Γ to *L* valleys in the conduction band. A certain construction of the curve at low energies is explained by the surface built-in electric field influence. At higher photon energies, this influence is overshadowed by the contribution of ballistic electron motion [24].

In a parabolic conduction band, the excess energy of photoexcited electrons is equal to

(5) $$\epsilon_{ex}\left(h\nu \right)=\frac{m_{hh}}{m_{hh}+m_{e}}\left(h\nu -\epsilon_{g}\right)$$

where $m_{hh}$ and $m_{e}$ are the heavy hole and electron effective masses, respectively. When transitions to *L* valleys begin, the duration of electron momentum relaxation decreases from ~200 fs to ~80 fs [25], so their ballistic movement ends earlier. In addition, electron transitions from the low-mobility *L* valleys back to the Γ valleys are much slower, taking about 3 ps [26], which results in an additional reduction of the photocurrent. All this suggests that the discussed maximum of THz excitation dependence on photon energy occurs due to the onset of intervalley electron scattering. Therefore, after deducting the intervalley phonon energy ($\hbar \omega_{iv}=$ 29 meV) from the $\epsilon_{ex}$ value at the photon energy that corresponds to TES maximum, we obtain the intervalley energy distance $\Delta \epsilon_{\Gamma L}=$ 0.3 eV. Based on similar considerations, we can determine the energy distance from the conduction band bottom to another group of additional valleys at *X* points of the Brillouin zone, which is evident in the sudden change in the inclination of the spectral dependence at hν= 1.92 eV, $\Delta \epsilon_{\Gamma X}=$ 0.42 eV.

In narrow-gap semiconductors, the surface THz emission has several specific features. As a rule, the surface electric field in these materials is weak, so the main reason for the generation of THz pulses is the spatial separation of electrons and holes due to their different velocities as they move away from the photoexcited surface. There is no THz emission when a semiconductor is illuminated with light whose photon energy does not exceed $\epsilon_{g}$. The emission starts when the excited electrons move into bulk a distance exceeding the photoexcited region width near the surface and the electric field caused by charge carrier separation appears. Let us assume that this width is ≈1/α, where α is the optical absorption coefficient of the material. Thus, the sufficient condition for emission of THz pulses is

(6) $$\nu \left(\epsilon_{ex}\right)\tau_{m}>1/\alpha$$

where $\nu (\epsilon_{ex})$ is the electron group velocity. In a two-band Kane band structure model [27] that is typical for narrow-gap III–V semiconductors, the conduction band dispersion relation is written as

(7) $$\frac{\hbar^{2}k^{2}}{2m_{e}}=\epsilon_{ex}\left(1+\beta \epsilon_{ex}\right)$$

where the electron effective mass at the conduction band edge me and the non-parabolicity factor β. The electron group velocity in this band can be expressed as

(8) $$\nu \left(\epsilon_{ex}\right)=\frac{\delta \epsilon_{ex}}{\hbar \delta k}=\frac{\sqrt{2\epsilon_{ex}\left(1+\beta \epsilon_{ex}\right)}}{\left(1+2\beta \epsilon_{ex}\right)\sqrt{m_{e}}}$$

**Figure 8:**
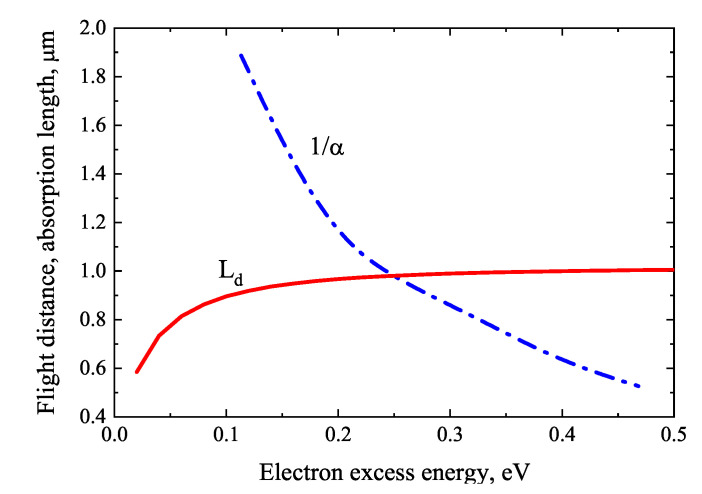
Quasi-ballistic flight distance (L_d_) and absorption length (1/α) as electron excess energy functions in InSb. Reproduced with permission from [23] © IOP Publishing.

Figure 8 compares the electron quasi-ballistic flight range and absorption length using InSb parameters taken from [28,29]. When calculating the excess energy of electrons, the heavy hole band is assumed to be parabolic. The comparison of both dependencies shows that the conditions for the appearance of THz emission are reached only when the electron excess energy exceeds 0.35 eV, almost twice the InSb energy bandgap at room temperature.

**Figure 9:**
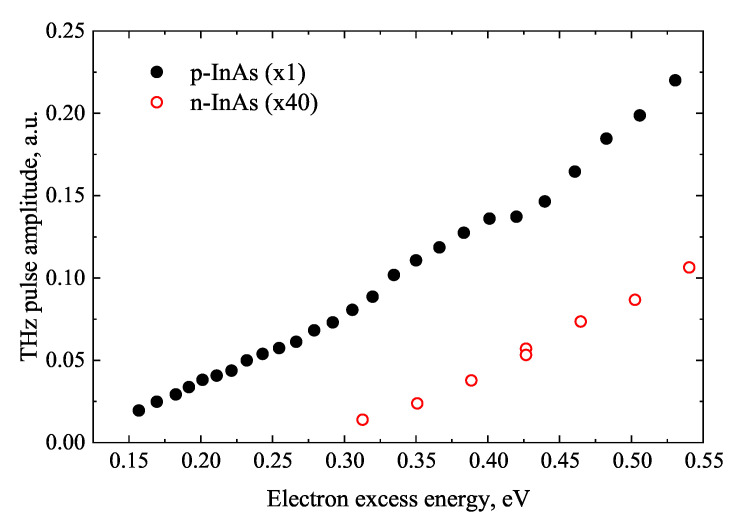
Initial parts of THz excitation spectra generated by p-InAs and n-InAs. Reproduced with permission from [23] © IOP Publishing.

A similarly delayed onset of THz emission is also characteristic for n-type InAs. However, in p-type InAs crystals, this process starts much earlier due to the strong inversion layer formed on the surface of this material and the resulting strong surface electric field [11]. The initial parts of the THz excitation spectra of these two materials are shown in Figure 9. It should be noted that the electric field existing on the p-InAs surface slows the electrons moving towards bulk, so its influence is manifested only by the distortion of electron trajectories and the increase of the lateral photocurrent components. When determining the conduction band subsidiary valley position in these semiconductors, it is also necessary to use the non-parabolic electron energy dispersion law, described by Equation (7).

**Figure 10:**
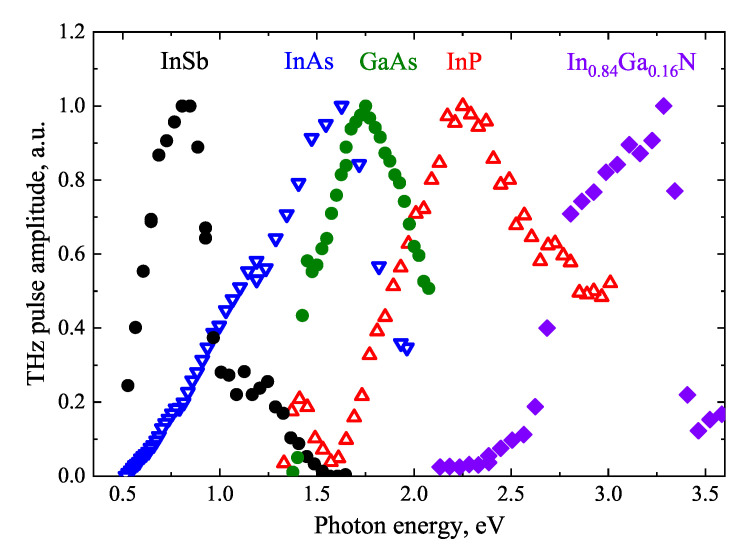
TES spectra of InSb [8,23,31], InAs [23,32,33], GaAs [23], InP [34], and In_0.84_Ga_0.16_N [30].

The THz excitation spectra of various A3B5 group semiconductors are plotted in Figure 10; the intervalley energy separation determined from such dependencies are presented in Table 1. It should be noted that the maximum pulse amplitude observed in InGaN is not due to the interlayer electron transfer [30]. This process in nitrides starts at photon energies exceeding those used in the experiment, and the observed decrease in THz pulse amplitude at hν≈ 3.5 eV is caused by the influence of the photocurrent excited in the heterojunction between GaN and a relatively thin (70 nm) InGaN layer.

## 4. Other Semiconductor Crystals

### 4.1. Germanium

**Figure 11:**
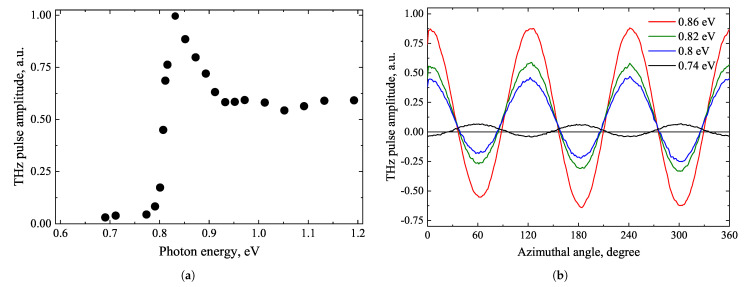
THz excitation spectrum of germanium (**a**), and azimuthal angle dependencies measured from a (111) cut sample at different laser photon energies in transmission geometry perpendicular to surface excitation (**b**). Reproduced from [39], with the permission of AIP Publishing.

Ge is an indirect bandgap ($\epsilon_{g}$ = 0.66 eV) semiconductor with valence band maximum located at the Γ points and conduction band minimum at the *L* points of the Brillouin zone. The direct bandgap of Ge at the Γ points corresponds to the energy of 0.8 eV [38]. The Γ minimum of the conduction band is located 0.14 eV while *X* minimum is at 0.19 eV above the *L* minimum. The dependence of generated THz pulse amplitude from unintentionally doped n-Ge crystal with ρ= 22 Ωcm resistivity and $1.5\cdot 10^{14}$ cm${}^{-3}$ electron density on the exciting photon energy is plotted in Figure 11a. The dependencies on the azimuthal angle, measured by illuminating the (111) crystal plane with a femtosecond laser beam of different wavelengths, perpendicular to the surface, are shown in Figure 11b [39]. As could be expected, a more pronounced THz emission is observed when the photon energy reaches the direct bandgap at Γ points.

Similarly to other semiconductor crystals described above, the amplitude of THz pulses emitted from laser-excited Ge surfaces depends on the angle between the optical field vector and crystalline axes—the azimuthal angle. Phenomenologically, such dependencies are described in terms of different-order optical nonlinearities. Since Ge is a centre-symmetrical crystal, the lowest available nonlinearities are of the third order, such as the EFIOR [40]. Microscopically, the presence of these effects in semiconductors is explained by the lateral surface photocurrents appearing due to an optical alignment and anisotropic electron energy dispersion law. It is suggested in [39] that the clear azimuthal angle dependencies are most probably caused by the warping of the heavy hole valence band [17] rather than the non-sphericity of the conduction band.

A rather unexpected behaviour of azimuthal angle dependence is observed when the Ge sample is excited with photon energies below the direct bandgap energy. Though, characteristic proportionality for (111) plane of sin3ϕ persists, the pulse phase polarity flips over. In [39] these observations are explained by the photoexcitation involving long wavelength intervalley phonons and the anisotropy of *L* valleys.

**Figure 12:**
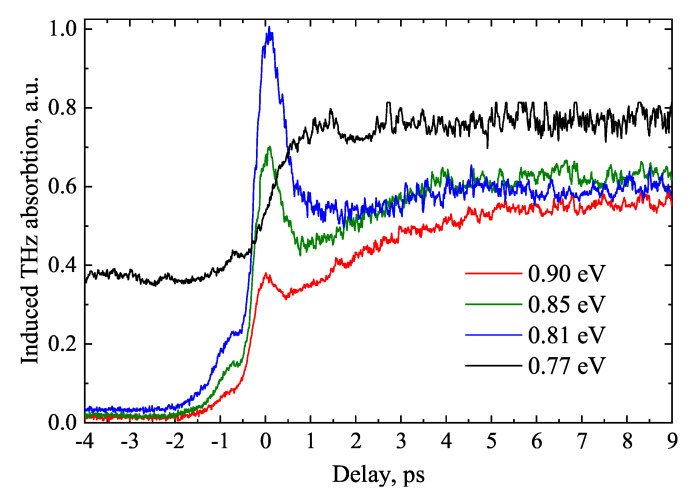
Optical pump—THz probe transients from Ge excited by different wavelength femtosecond pulses at excitation fluency of around 1 μJ/cm^2^. Reproduced from [39], with the permission of AIP Publishing.

When the crystal is excited by photons of various energies, the electron dynamics measured at longer times than THz emission duration are also drastically different. The optical pump—THz probe experiment were used for this study (Figure 12). These dynamics, which are typically used to study electron lifetimes, vary from slowly changing with a high background level when hν= 0.77eV, through a sharp THz absorption peak when excited by photons whose energy corresponds to TES maximum, to the gradual disappearance of this peak at highest photon energies. The last two dependencies are explained by the excitation of electrons into the high-mobility Γ valley and their scattering into the low-mobility *L* and *X* valleys. Scattering into the latter valleys is the fastest, so the initial peak of the THz absorption is not fully visible due to the limited temporal resolution of the experiment. Meanwhile, the significantly different dependence corresponding to hν= 0.77 eV can be explained in two circumstances. First, the slower indirect electron jumps from valence band to conduction band *L* valleys, which require the participation of phonons. Second, the optical absorption coefficients differ by two orders of magnitude in cases of direct and indirect transitions. Direct transitions excite electrons in a thin layer near the crystal surface, while indirect transitions excite electrons almost homogeneously throughout the entire volume of the sample, so different signal dynamics indicate different recombination rates of bulk and surface charge carriers. The observed high background level indicates that the electron recombination time in the bulk is longer than the repetition rate of optical pulses that is equal to 5 μs.

### 4.2. Tellurium

Tellurium (Te), a p-type narrow bandgap semiconductor material has recently attracted much attention due to its peculiar band structure, spin properties, and high-efficiency thermoelectric performance. It has a distinctive place among materials investigated by surface THz emission. Although the intensity of THz radiation emitted from the surface of this crystal is approximately two orders of magnitude lower than that from p-type InAs, the research on tellurium has highly contributed to the understanding of underlying physics of this observation. Two different physical mechanisms leading to the surface emission of THz waves were proposed when investigating Te crystals: THz electromagnetic wave emission by coherent infrared active phonons and due to the ultrafast buildup of photo-Dember field [13].

**Figure 13:**
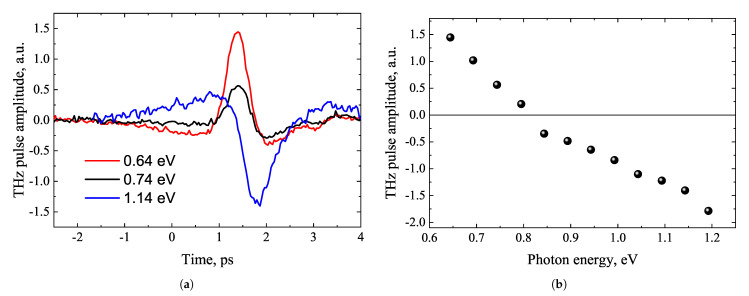
THz pulses excited from Te (001) surface at different photon energies (**a**), and THz excitation spectrum of the same sample (**b**). Reproduced from [41], with the permission of AIP Publishing.

**Figure 14:**
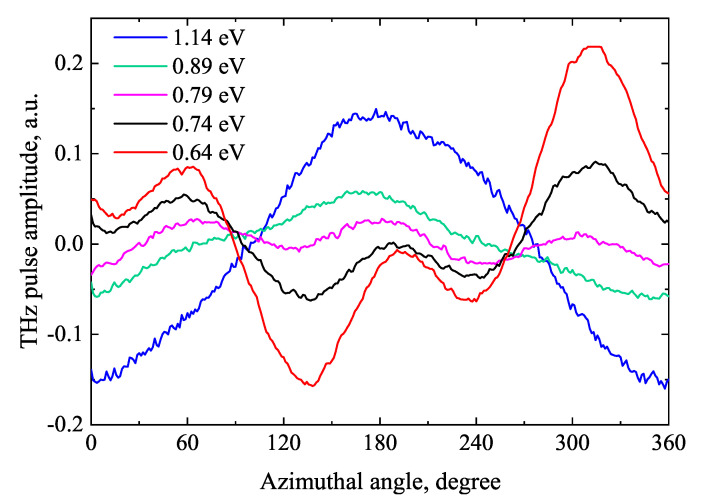
THz pulse amplitude azimuthal angle dependencies at different P-polarized femtosecond optical beam photon energies. The Te (001) sample is illuminated to surface normal. Reproduced experiment from [41] at more photon energies.

THz excitation spectra of Te crystals were first measured in ref. [41]. They revealed several features not observed in other semiconductors. The most important of them was the polarity inversion of THz pulses in the range of photon energies 0.8–0.85 eV (Figure 13). In the areas where the photon energy was lower and higher than these values, the dependence of the THz signal amplitude on the azimuthal angle also differed significantly (Figure 14). In the range of lower photon energies, they resembled a combination of cosϕ and cos3ϕ functions, while at higher photon energies they were proportional to cosϕ. It should be noted that all these experiments were performed by illuminating the samples with optical beams parallel to the surface normal. The THz radiation exiting the crystal was recorded in reflection direction. In such an experimental geometry, only the THz radiation caused by the lateral photocurrent components can be observed. These components can occur for several reasons, in ref. [41] named as such the transverse photo-Dember effect at high photon energies [42] and the lateral ballistic movement of charge carriers at low photon energies arising due to the energy band anisotropy.

The latter component is characterized by cos3ϕ proportional azimuthal angle dependencies and probably has a similar origin as in other semiconductors [17]. Phenomenologically, they are described in terms of second or third-order optical nonlinearities; their microscopical origin stems from the anisotropy of dynamic photoconductivity caused by ballistically moving optically-aligned carriers. Such non-linear effects were also observed in tellurium before; they were interpreted as the optical rectification [43] or the photogalvanic effect [44].

On the other hand, when the surface of an anisotropic semiconductor is exposed to light that is strongly absorbed, a carrier-concentration gradient normal to the sample surface is formed and the diffusion of excess carriers into the bulk starts. Due to the carrier effective mass anisotropy, however, the diffusion of electrons generally proceeds in a direction different from that of holes. Because of this, an electric current flows and a dynamic polarization can develop along the surface. The radiating dipole has a built-in direction in the sample and is rotating with it resulting in a cos3ϕ—type azimuthal angle dependence. In addition, photons with energy greater than 0.9 eV can excite electrons to such an extent that they are scattered into the high effective mass subsidiary conduction band valleys and their mobility becomes lower than the mobility of holes. The excess energy of electrons excited from the second, larger hole-mobile valence H5 band calculated for parabolic energy bands and carrier effective masses taken from [45] is equal to 0.33 eV, thus greater than the energy distance to the high effective mass valley at point A of the Brillouin zone, the value of which is close to 0.3 eV as it is evidenced by the anomalous Hall-effect [46] and Gunn-effect [47] research. This explains the polarity inversion of THz pulses at high photon energies.

However, the aforementioned spectral dependencies of tellurium are not yet completely clear. Recent studies of energy bands of Te [48,46] show that branches with low effective mass and high anisotropy exist in the valence band of tellurium. Electron transitions from these lower branches may provide an alternative explanation for the THz pulse polarity inversion and the complex azimuthal angle dependencies observed when THz radiation is excited in different crystal planes.

### 4.3. Gallium Selenide

GaSe is a group A2B6 crystal with its layers connected by a weak van der Waals force. It is a birefringent optical material with a strong nonlinear optical response in the infrared range. GaSe is often used as a nonlinear medium for THz wave emission [49,50], electrooptic sampling [51], and frequency comb generation [52]. When photoexcited by 10 fs duration and 780 nm wavelength laser pulses, GaSe radiates terahertz radiation pulses with frequencies up to 41 THz via optical rectification mechanism [53]. The energy band gap of GaSe is as large as $\epsilon_{g}\approx$ 2 eV, therefore it can be an attractive material for photodetectors operating in the visible range [53]. Nevertheless, the semiconducting properties of GaSe have not been studied in sufficient detail so far. There is no consensus on the exact value of the energy bandgap and on what is the energy separation between the main Γ valley and the additional *M* valleys in the conduction band. The size of this gap, determined by different authors, varies from −0.13 eV [54] to +0.3 eV [55].

**Figure 15:**
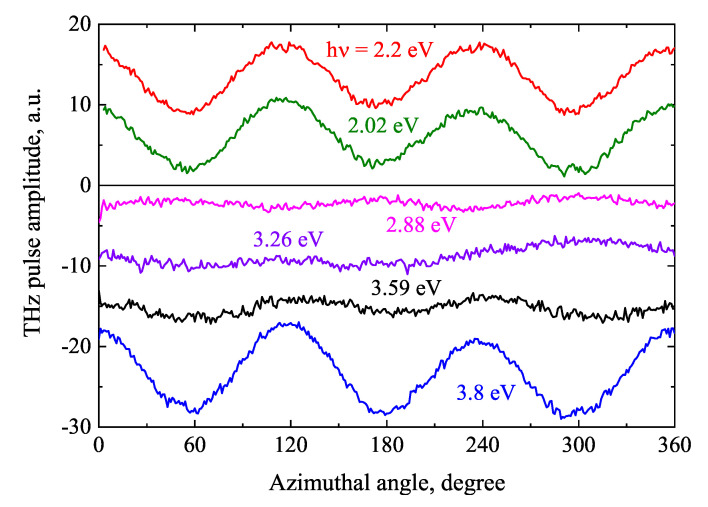
Azimuthal angle dependencies of THz pulse amplitude at different P-polarized femtosecond optical beam photon energies. The GaSe sample is illuminated at a 30angle to surface normal. Reproduced from [56], with the permission of AIP Publishing.

**Figure 16:**
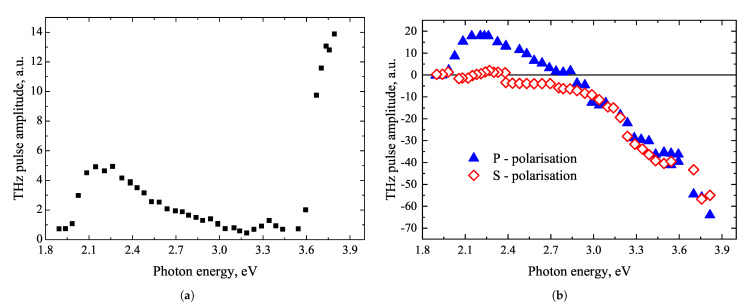
TES spectrum of GaSe azimuthal angle dependent THz pulse part (**a**), and the azimuthal angle independent part when illuminated by S- (red squares) and P-polarized (blue triangles) optical beams (**b**). Reproduced from [56], with the permission of AIP Publishing.

THz pulses emitted from GaSe excited by laser pulses with photon energies higher than the energy bandgap were first measured in ref. [56]. A characteristic feature of this material with a trigonal crystal structure is the pronounced dependence of the signal on the azimuthal angle (Figure 15) and different, mutually independent spectral dependencies of the THz signal components dependent and independent of this angle. The excitation spectra of both of these components are presented in Figure 16, respectively. Spectral THz pulse excitation experiments with optical pulses lower and slightly higher than $\epsilon_{g}$ photon energies were performed by D. Zhai et al. [57]. This work observed two THz emission peaks: a first peak at hν= 1.77 eV, where phase matching of the optical and THz beams is realized, and a second in the vicinity of the $\epsilon_{g}$ (hν= 2.205 eV) that can be attributed to the resonance of GaSe nonlinearity. The first peak dominated in thick GaSe layers comparable to the coherence length (~1 mm); in layers thinner than 0.1 mm, the second peak prevailed.

All studies in the work of Norkus et al. [56] were performed using 50 μm thick GaSe samples, where the influence of the nonlinear optical response of bound electrons was minimal. As for the azimuthal angle ϕ-independent component, it is logical to assume that it is due to the dynamic photocurrent flowing from the surface to the bulk. In the part of the spectrum between 2 eV and 3 eV, this current appears due to electron transitions from the highest valence band at the $\Gamma_{v1}$ point. Such transitions are only possible when the optical field is parallel to the c-axis, so the signal is observed only after illuminating the c-plane with a p-polarized optical beam impinging at an incline angle. The most likely reason for the maximum observed at hν= 2.4 eV is the electron transitions from the Γ minimum of the conduction band to the subsidiary *K* valleys; from its position, the energy of those valleys can be determined. When hν> 3 eV, the excitation of electrons from the second valence band $\Gamma_{v2}$ begins. Such excitation is possible when the optical field is perpendicular to the c axis, so a component independent of ϕ is observed for both light polarizations. Polarity inversion of THz pulses probably occurs as in the case of Te due to the fact that the effective masses of photoexcited holes and their velocities are higher than those of electrons. The anisotropy of the second valence band can also be the reason for the large azimuthal angle-dependent THz pulse amplitude component observed for hν> 3 eV. Meanwhile, the weaker maximum of this component at the optical absorption edge could be caused by the resonant enhancement of the optical rectification effect described in ref. [57].

## 5. Semiconductor Nanostructures

The TES method described in this article does not require any electrical contacts attached to the sample, so it is ideal for objects on which such contacts are difficult to fabricate. It concerns various semiconductor nanostructures: ultra-thin layers, nanowires, quantum dots, etc. Several studies of such structures is reviewed in this section.

### 5.1. Bismuth Layers

Bi is a semimetal that has many extraordinary characteristics because of its high electron energy band structure anisotropy, small electron and hole effective masses, and high mobilities [58]. In addition, Bi is the first material in which the electron quantum confinement effects were demonstrated [59]. A two-dimensional (2D) carrier confinement occurs in the semimetal—semiconductor transition in thinner than ~30 nm Bi layers [60]. Quantum confinement effects are also observed in Bi nanowires [61] and nanoparticles [62]. TES can be a useful tool for studying the properties of these materials.

THz excitation spectra were studied from thin bismuth layers fabricated using two different technologies: electrochemical deposition on noble metals [63] and molecular beam epitaxy (MBE) on silicon substrates [64]. The thickness of bismuth layers was from 50 nm to 600 nm in the first case and as thin as 7 nm in the second case. Electrodeposited layers were polycrystalline, their structure and properties depended little on the substrate.

**Figure 17:**
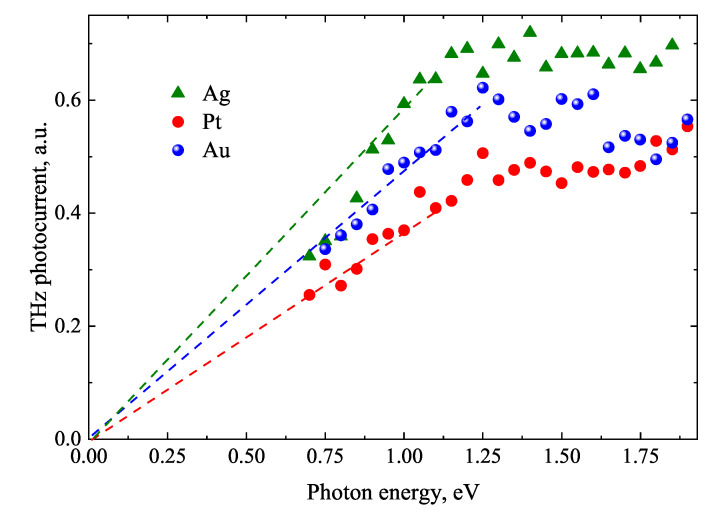
Generated THz photocurrent as a function of exciting photon energy. Three 100-nm-thick bismuth films electrochemically deposited on different substrates [63].

Figure 17 shows TES spectra measured from 100 nm thick Bi layers grown on three different noble metals. The peak values of the photocurrent transients obtained after integrating THz pulses rather than THz pulse amplitudes were plotted on this graph. All the curves are characterized by saturation when photon energy exceeds 1 eV; interpolation to the low photon energies leads to 0 eV, testifying the semimetallic nature of Bi. Since the energy band bending and built-in electric fields in semimetallic Bi are absent, the most probable cause of ultrafast photocurrents and THz pulse emission is the photo-Dember effect. The thermalization of photoexcited carriers in Bi due to intense electron-electron scattering occurs within a few tens of femtoseconds—faster than their interaction with phonons [65], so the influence of ballistic electron motion can be ruled out.

**Figure 18:**
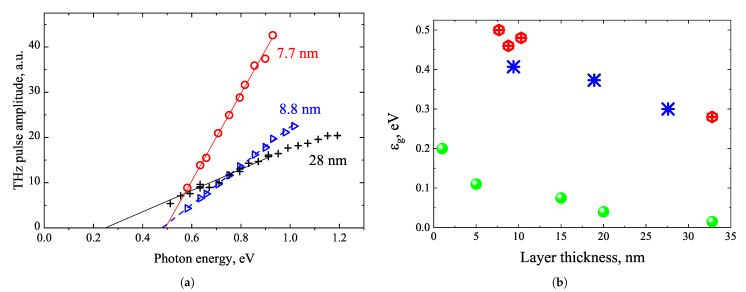
TES spectra of three different thickness MBE grown β-Bi layers (**a**). The photon energies at which the THz emission sets on as a function of layer thickness, where hexagons are β-Bi, stars are α-Bi, and full circles are indirect bandgaps of Bi (**b**). Reproduced from [66], with the permission of AIP Publishing.

The THz emission characteristics of Bi samples grown by MBE technology on (111)-oriented silicon substrates were quite different [66]. Depending on growth conditions, bismuth layers of two phases can be obtained: Bi(110) (α-Bi) or Bi (111) (β-Bi) layers. β-Bi layers were characterized by hexagonal crystal symmetry, which was mirrored in clear THz signal amplitude dependencies on azimuthal angle having the cos3ϕ shape curves. In addition, the THz excitation spectra measured in layers of different thicknesses allowed to reliably determine their direct energy bandgaps (Figure 18a). The direct bandgap dependence on layer thickness in Figure 18b is compared with the indirect bandgaps as found from the optical [67] and temperature-dependent electrical [68] measurements.

### 5.2. InAs Nanowires

InAs is known as a narrow bandgap semiconductor ($\epsilon_{g}\approx$ 0.36 eV) with a small electron effective mass, very high electron mobility (>20 000 cm^2^/Vs at 300 K) and unique electro-optical properties [69]. It could be used as a material for quantum wells and quantum dots, as well as for applications in high-speed electronic and mid-infrared optoelectronic components. This is, however, hampered by large epitaxial strains and defect densities arising due to the significant crystal lattice mismatch (~11.6 %) with Si [70]. The reduction of these critical lattice matching requirements is shown to be possible by growing free-standing III–V compound semiconductor nanowires on Si substrates via the vapour-liquid-solid (VLS) growth method [71].

Electrical measurements conducted on nanowires to determine charge-carrier mobility are often obscured by properties of the electrical contacts, therefore, the contactless THz techniques are ideal means for their characterization. The optical pump-THz probe technique is successfully used for the investigation of photoconductivity and carrier lifetimes in InP [72] and GaAs [73] nanowires. The THz excitation spectroscopy is, for the first time, applied to NW characterization in [74].

**Figure 19:**
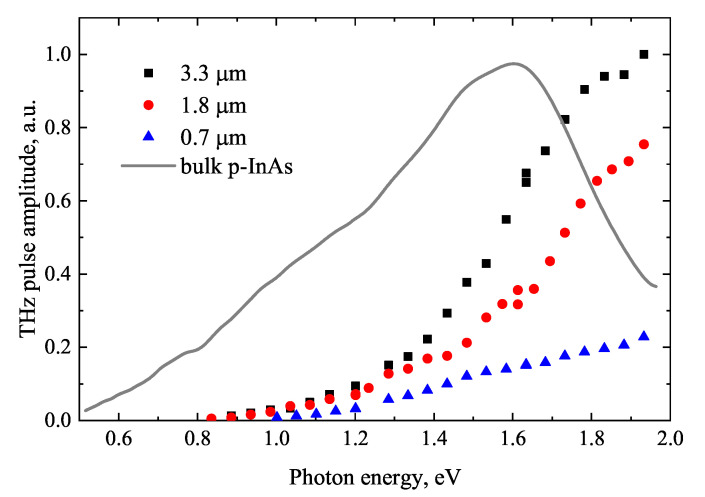
THz excitation spectra obtained from different length InAs nanowires and bulk p-InAs as reference. Adapted with permission from [74]. Copyright 2014 American Chemical Society.

The THz excitation spectra measured from three different length InAs NW samples are shown in Figure 19 together with the bulk p-type InAs dependence. Several distinct differences in the results obtained from bulk and NW samples are evident. No spectral maximum typical for the majority of bulk III–V semiconductors is observed in the investigated photon energy range and the emission from the NW arrays sets on at higher photon energies. These differences are most likely caused by the lower electron mobilities in intrinsic non-passivated InAs NWs due to surface effects that dominate the charge carrier transport properties. Generally, electron mobilities in InAs NWs with diameters from 70 to 150 nm, such as those investigated in [73], are in the range of ~800–2000 cm^2^/(Vs) [75], which are much lower than in bulk InAs. Therefore, the electron momentum relaxation time $\tau_{m}$ can be comparable or even shorter than intervalley scattering times and electron transitions to subsidiary conduction valleys should only have a minor effect resulting in the absence of TES maximum.

**Figure 20:**
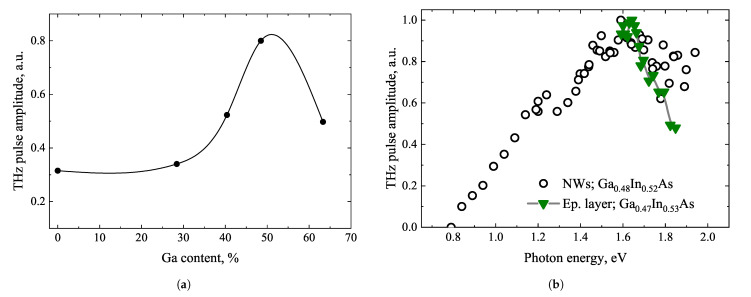
THz pulse amplitudes emitted from GaInAs NWs when normalized to bulk p-InAs amplitude (**a**). THz excitation and photoluminescence spectra of Ga_0.47_In_0.53_As NWs (**b**). Reproduced from [77], with the permission of AIP Publishing.

NW properties at THz frequencies can also be strongly influenced by the surface structure of InAs. It is a well-known fact that this material has a ~100 nm surface electron accumulation layer with intrinsic electron density in the order of 10^17^ cm^−3^[76]. Surface accumulation and related electron scattering can be reduced by alloying InAs with gallium. The THz radiation from five Ga_x_In_1−x_As NWs of different compositions is studied in [77]. It is found that the amplitude of THz pulses increases with increasing Ga fraction and reaches a maximum at x ≈ 0.47 (Figure 20a). In addition, a maximum in the THz excitation spectra is observed from NWs with close Ga and In compositions (Figure 20b). It should be noted that NW arrays with intermediate compositions, i.e., Ga-content 0.4–0.5, are found to radiate THz pulses much more efficiently (THz amplitudes higher by a factor of 2–3) as compared to InAs NWs. When considering the fill factor of NW arrays, the THz generation from Ga-rich GaInAs NWs translates to ~4 times stronger emission than that from bulk p-type InAs.

## 6. Heterostructure Band Offsets

The TES technique assumes ballistic propagation of photoexcited carriers in semiconductor surfaces after femtosecond laser pulse excitation. This propagation can last for a comparable time to that of the pulse relaxation $\tau_{m}$; due to the spatial separation of electrons and holes, the dynamic electric dipole and the emitted THz pulse amplitude is greater the further the electrons move into the bulk. Thus, this technique can be used to study electron states further from the surface. One such application can be the determination of energy band offsets in heterojunctions between two different materials.

**Figure 21:**
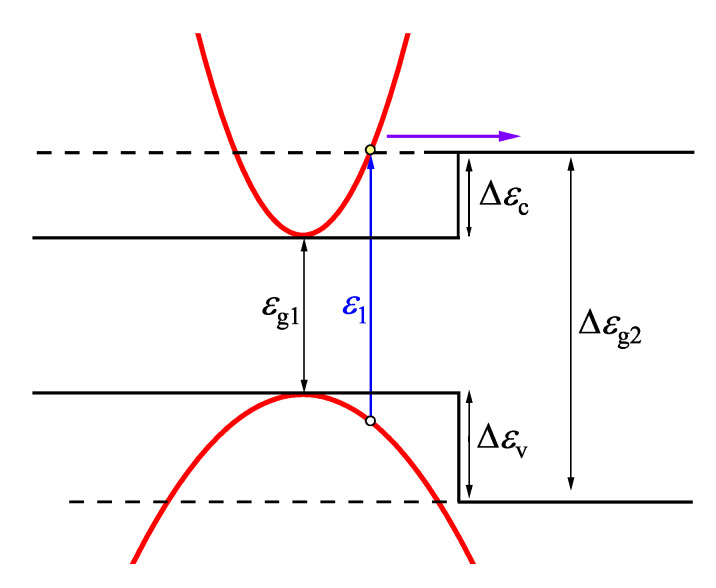
Scheme of optical transitions that correspond to the threshold photon energy $\epsilon_{1}$ for THz emission criterion. The THz pulse is emitted when electron excess energy is sufficient to overcome the $\Delta \epsilon_{c}$ potential barrier. Reprinted with permission from [9] © Optica Publishing Group.

Displacements of the conduction and valence band levels, $\Delta \epsilon_{c}$ and $\Delta \epsilon_{v}$, may occur at a heterojunction border consisting of two semiconductors with different energy bandgaps. They would act as energy barriers for moving electrons and holes, thus affecting the performance parameters of all devices containing such junctions. A number of theoretical and experimental works have been devoted to the determination of these important parameters; those works are summarized in a review [78]. The terahertz excitation spectroscopy as a new way of estimating the energy band offsets is proposed in the paper [9]. If the layer with a narrower energy band gap is thin enough and on top of the sample, after illuminating it with femtosecond optical pulses, stronger THz emission would start only when the excess energy of excited electrons exceeded the energy offset in the conduction band (Figure 21). This allows to directly determine the energy band line-up in the heterostructure from TES measurements.

Therefore, the THz emission is expected at photon energies equal to and above

(9) $$h\nu =\epsilon_{1}=\epsilon_{g}+\Delta \epsilon_{c}\left(1+\frac{m_{e}}{m_{h}}\right)$$

Here $\epsilon_{g}$ is the bandgap of the top layer semiconductor. In a real experiment, the THz emission edge is smeared for two reasons: the convolution with the finite Gaussian spectrum of the laser pulse, and the energy-dependent transmission coefficient of the potential barrier $T(\epsilon_{⊥})$

(10) $$T\left(\epsilon_{⊥}\right)=\frac{4\sqrt{1-U/\epsilon_{⊥}}}{{\left(1+\sqrt{1-U/\epsilon_{⊥}}\right)}^{2}}$$

where $\epsilon_{⊥}$ is the electron kinetic energy and U $=\Delta \epsilon_{c}$ is the potential barrier that corresponds to the conduction band offset. It has been shown in [9] that at the energy $\epsilon_{1}$, the THz emission onset can be approximated by the exponential function $A\left(h\nu \right)={\left(h\nu -\epsilon_{1}\right)}^{3/2}$.

**Figure 22:**
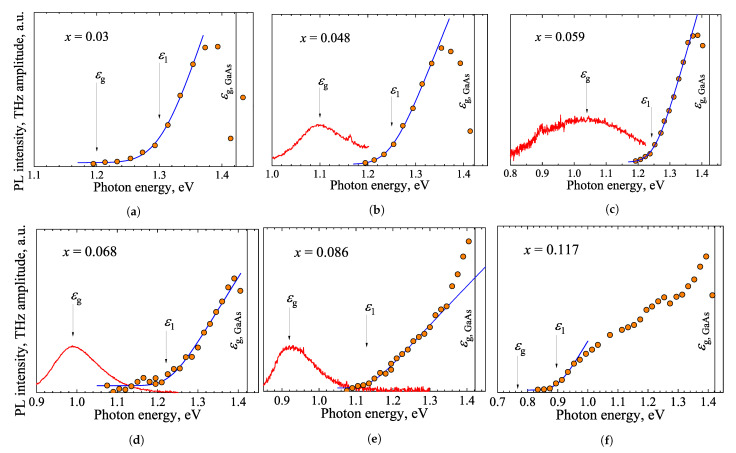
(**a**–**f**) TES spectra (orange dots) of investigated GaAs_1−x_Bi_x_-GaAs heterostructures with different Bi concentrations. Red curves are photoluminescence spectra. Blue curves correspond to theoretical spectral shapes A(hν) of THz emission onset. Reprinted with permission from [9] © Optica Publishing Group.

Figure 22 shows the results of TES measurements performed on several GaAs_1−x_Bi_x_-GaAs heterostructures. The energy band line-ups in this heterojunction were investigated in several previous studies. The conduction band offset equal to nearly 40 % of the energy band gap difference of GaAs and GaAsBi has been predicted theoretically in [79]; the value of 23% was found from the X-ray photoemission [80] and as high as 48% from the photoreflectance studies [81]. Additional uncertainty was introduced by the photoluminescence investigations from multiple quantum wells in ref [82]. Authors concluded that the arrangement of bands in the GaAsBi-GaAs heterojunction corresponds to the staggered (type-II) and not to the straddling (type-I) variant. It was shown by [9] that the conduction band offset in this heterojunction can be found by matching the threshold energy $\epsilon_{1}$ of the TES spectrum with the A(hν) function. These studies provided a 45% offset value at 11% Bi concentration.

**Figure 23:**
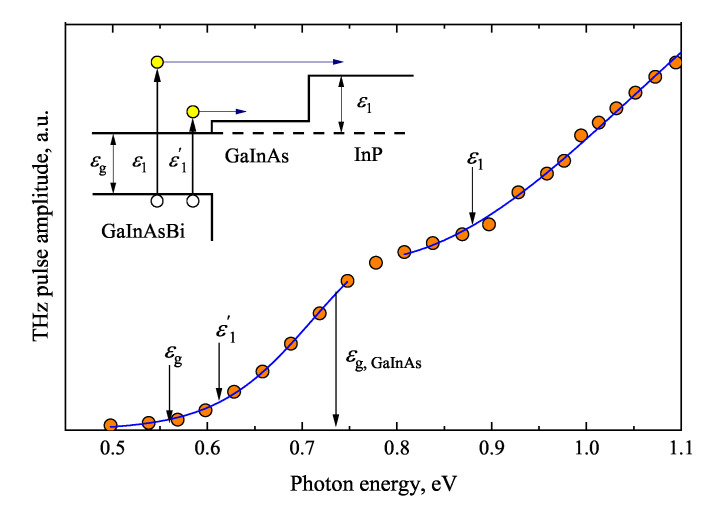
THz excitation spectrum and energy diagram (inset) of a bismide heterostructure containing a 150 nm thick GaInAsBi layer (3.5% Bi), 250 nm Ga_0.47_In_0.53_As layer, and InP substrate. Adapted from [83].

Similar measurements are also performed on various GaInAs and GaInAsBi heterojunctions grown on InP substrates [83]. Figure 23 shows the TES spectrum measured from a double heterojunction with layers of both these materials. From this graph, the relative conduction band offsets $Q=\Delta \epsilon_{c}/\Delta \epsilon_{g}$ of GaAsInBi-InP heterojunction as well as the GaAsInBi-GaInAs heterojunction are visible. The determined *Q* parameter of the lattice-matched GaInAs-InP heterojunction is equal to 38%, which agrees with the recommended average value of 43% [84] within 10% error. For the strained Ga_0.47_In_0.53_As_1−x_Bi_x_-InP heterostructure, the relative conduction band offset is smaller and it reduces to 34% at 6% bismuth concentration.

**Figure 24:**
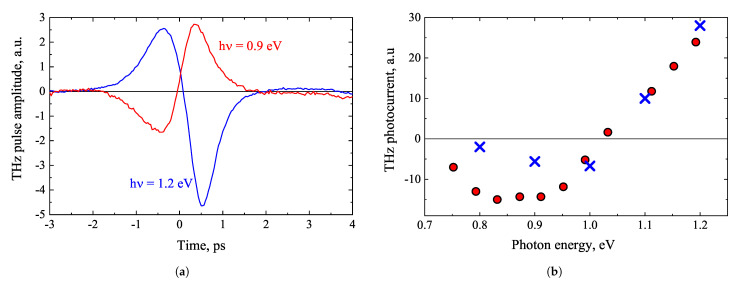
THz electric field pulses measured at two different optical wavelength beams impinging on GaInAs-InP heterostructure from the InP substrate side (**a**), and corresponding THz excitation spectrum (full red circles). Blue crosses show the Monte Carlo simulation results (**b**) [85].

Quite informative TES spectra are also obtained by exciting the semiconductor heterojunction from the wide bandgap layer side. Such measurements are performed on GaInAs and GaInAsBi layers grown on InP substrates [85]. The results obtained from the GaInAs-InP heterostructure are illustrated in Figure 24. A characteristic feature of these results is the THz pulse polarity inversion at photon energies >1 eV. This feature could be explained as follows. When photon energy is low and electrons with excess energy below the conduction band offset $\Delta \epsilon_{c}$ are excited in the narrow-gap part of the heterostructure, the photocurrent arises as a result of electron reflections from epitaxial layer’s interface and it is directed towards the surface. At higher photon energies, when the excess energy becomes higher than *U* and it is sufficient enough to overcome the potential step, the electrons moving towards the substrate also take part in the net photocurrent. This photocurrent is of opposite direction in comparison to that flowing in the top epitaxial layer. At photon energies exceeding the threshold energy $\epsilon_{g}+\Delta \epsilon_{c}$, the photocurrent of electrons transmitted into substrate is higher than that in the epitaxial layer, which results in the polarity inversion of a THz pulse. This is confirmed by the Monte Carlo simulation results shown in Figure 24b. One important outcome of this study is the realization of optics-like effects in electronic systems. Ballistic electrons are coherent de Broglie waves that can be reflected or refracted at heterointerfaces between different semiconductors, much like electromagnetic waves [86,87]. The trajectories of ballistic electrons entering from narrow-gap to wide-gap semiconductor are refracted towards heterojunction, so the lateral current component they create and the generated THz pulse amplitude increases. These results can also be considered as additional evidence of the ballistic electron motion that generates THz pulses from semiconductor surfaces.

## 7. Conclusions and Insights for the Future

Today, table-top generation of ultrashort THz pulses from semiconductor surfaces photoexcited by femtosecond optical pulses with photon energies in the range from 0.5 eV to more than 3 eV is now possible in a routine and reliable manner. Since the surface THz emission effect is in most cases caused by the directional and quasi-ballistic movement of photoexcited electrons into the semiconductor bulk, it is a versatile tool for investigating the electron energy band structure and hot-electron characteristics.

Terahertz excitation spectroscopy has already been used to determine the positions of various energy dispersion law extrema in A3B5 group compounds and other semiconductors. In some materials, e.g., InAs, the conduction band intervalley energy separation has been determined experimentally for the first time with TES technique. This method made it possible to determine the details of electron energy spectrum in a layered GaSe crystal, and to measure the direct energy gap dependence on mono-elemental Bi layer thickness. One can expect that the TES measurements performed on semiconductor samples placed in magnetic field would provide additional information about charge carriers’ physical parameters such as the crystal symmetry influence on them. These kind of investigations would be valuable for studying topological insulators or magnetic structures.

It has been shown that the THz excitation spectroscopy is an excellent tool for determining the energy band offsets in semiconductor heterostructures. THz pulses generated by various wavelength femtosecond optical beams can also be exploited to characterize the properties of separate regions of multilayer semiconductor structures, e.g., the photovoltaic performance of multijunction solar cells [88].

As a contactless experimental technique, THz excitation spectroscopy is particularly useful for studying the electrical properties of various semiconductor nanostructures and layered Van der Waals bonded materials. The initial studies of these materials with TES methodology made it possible to estimate the direct bandgap dependence of bismuth layers on their thickness, the influence of surface electron accumulation on GaInAs nanowires’ electrical properties, and the formation of dark excitons in layered transition metal dichalcogenide samples [89]. TES with improved sensitivity and spatial resolution can become the main electrical and optical characterization technique of nanostructures and electronic and photonic circuits based on them.

## Figures and Tables

**Table 1:** Band structure parameters obtained from TES method. $\epsilon_{g}$ is the typical bandgap found in literature, $\epsilon_{0}$ is the TES onset, $\epsilon_{max}$ is the TES maximum, $\epsilon_{\Gamma L}$ is the energetic distance between Γ and *L* valleys.

Semiconductor	$\epsilon_{g}$, eV	$\epsilon_{0}$, eV	$\epsilon_{\max}$, eV	$\epsilon_{\Gamma L}$, eV
InSb [8,23,31]	0.18	<0.5	0.8	0.51
GaSb [35]	0.6	0.72	0.85	0.11
InAs [23,32,33]	0.35	0.5	1.55	1.08
GaAs [23]	1.42	1.42	1.76	0.3
Ga_0.43_In_0.57_As [36]	0.74	0.74	1.55	0.75
GaAsBi 8% Bi [37]	0.9	0.9	1.42	0.4
InP [34]	1.34	1.34	2.3	0.75

## Data Availability

No new data were created or analyzed in this study. Data sharing is not applicable to this article.

## Abbreviations

The following abbreviations are used in this manuscript:

TES	Terahertz Excitation Spectroscopy
